# HumanLectome, an update of UniLectin for the annotation and prediction of human lectins

**DOI:** 10.1093/nar/gkad905

**Published:** 2023-10-27

**Authors:** Boris Schnider, Yacine M’Rad, Jalaa el Ahmadie, Alexandre G de Brevern, Anne Imberty, Frederique Lisacek

**Affiliations:** Proteome Informatics Group, SIB Swiss Institute of Bioinformatics, CH-1211 Geneva, Switzerland; Computer Science Department, University of Geneva, CH-1227 Geneva, Switzerland; Proteome Informatics Group, SIB Swiss Institute of Bioinformatics, CH-1211 Geneva, Switzerland; Computer Science Department, University of Geneva, CH-1227 Geneva, Switzerland; Proteome Informatics Group, SIB Swiss Institute of Bioinformatics, CH-1211 Geneva, Switzerland; Computer Science Department, University of Geneva, CH-1227 Geneva, Switzerland; University Grenoble Alpes, CNRS, CERMAV, F-38000 Grenoble, France; Université Paris Cité and Université de la Réunion and Université des Antilles, INSERM, BIGR, DSIMB Bioinformatics Team, F-75014 Paris, France; University Grenoble Alpes, CNRS, CERMAV, F-38000 Grenoble, France; Proteome Informatics Group, SIB Swiss Institute of Bioinformatics, CH-1211 Geneva, Switzerland; Computer Science Department, University of Geneva, CH-1227 Geneva, Switzerland; Section of Biology, University of Geneva, CH-1205 Geneva, Switzerland

## Abstract

The UniLectin portal (https://unilectin.unige.ch/) was designed in 2019 with the goal of centralising curated and predicted data on carbohydrate-binding proteins known as lectins. UniLectin is also intended as a support for the study of lectomes (full lectin set) of organisms or tissues. The present update describes the inclusion of several new modules and details the latest (https://unilectin.unige.ch/humanLectome/), covering our knowledge of the human lectome and comprising 215 unevenly characterised lectins, particularly in terms of structural information. Each HumanLectome entry is protein-centric and compiles evidence of carbohydrate recognition domain(s), specificity, 3D-structure, tissue-based expression and related genomic data. Other recent improvements regarding interoperability and accessibility are outlined.

## Graphical Abstract

**Graphical Abstract:**
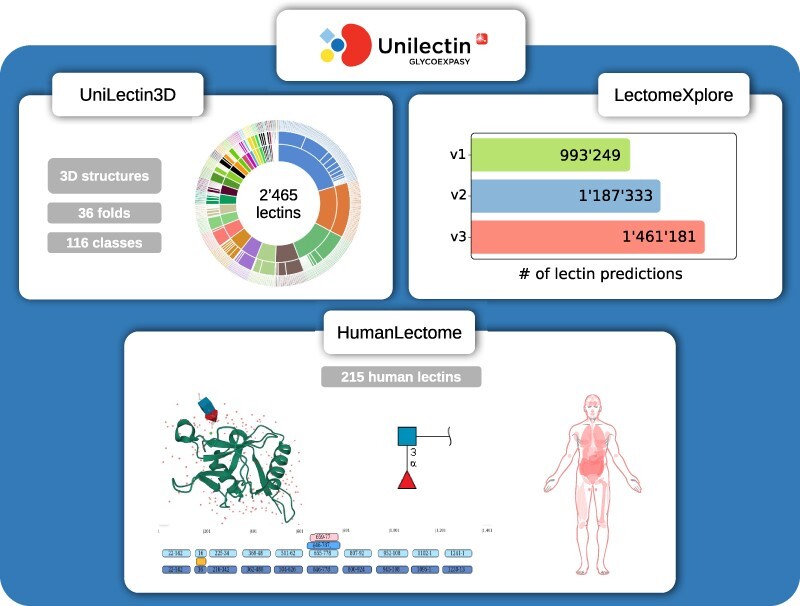

## Introduction

Lectins are a diverse group of proteins that are found in all organisms, including viruses, bacteria, plants, fungi and animals (1). Lectins are characterized by the presence of at least one Carbohydrate Recognition Domain (CRD) that reads the ‘glycocode’ displayed by glycolipids, glycoproteins or polysaccharides on all cell surfaces (2). Lectins play crucial roles in many biological processes that include quality control in glycoprotein biosynthesis (3), communication between cells in organisms (4), as well as self/non self recognition (5). Lectin-carbohydrate interactions are also involved in a large number of pathologies and lectins are therefore targets for drug design (6). Finally, their capacity to specifically recognize complex carbohydrates (also designated as glycans) and glycoconjugates, make lectins useful in biotechnology as biomarkers, biosensors or in drug delivery (7).

Knowledge of lectin 3D structures is important for understanding the protein CRD/glycan specificity as well as gaining information on the functional role of the whole protein (8). Interactions between carbohydrates and amino acids include hydrogen bonds, electrostatic bonds for charged sugars, van der Waals contacts, including some where aromatic residues uniquely interact with CH bonds of carbohydrates (9), and frequently involve bridging water molecules of calcium ions (10). Structural investigations also bring information on the oligomeric state of the CRDs that generate strong avidity of multivalent glycans, compensating the weak affinity at each CRD site. Collecting all available information on lectin 3D structures was the aim of UniLectin3D, the first module of the UniLectin portal (11,12).

The 3D structures of more than 2000 lectins in UniLectin3D served as the basis of a hierarchical definition of 107 classes built on 35 protein folds. This robust classification led to defining Hidden Markov Model (HMM) profiles (13) that were then used to screen sequence databases, namely NCBI-nr (14) and UniProt (15). This resulted in creating a second module called LectomeXplore (16). It now contains 1.4 million lectin predictions across all kingdoms. Additional modules were also proposed with the goal of tackling the issue of multivalency in some lectins (17). In several classes, the occurrence of tandem repeats in amino acid sequences hampers the design of HMM signatures (18). Modules were created to address this problem for two lectin folds, the HMM profiles of which were defined at the peptide repeat level. PropLec, dedicated to the β-propeller fold (already included in the 2021 update) and more recently, TrefLec focused on the β-trefoil folds, were included in UniLectin. Both were successfully used for the identification and further characterization of lectins with novel structural and functional properties (19,20). Two other modules were integrated since the 2021 description of UniLectin. MycoLec, devoted to lectins predicted in more than a thousand genomes of filamentous fungi and yeasts (21) and BiotechLec, an interactive table intended as a practical guide for lectin users in biotechnology (22). The present update briefly introduces these recent inclusions but mostly dwells on the latest module developed as a reference for the human lectome.

The need for developing a database dedicated to human lectins is justified by the biological importance of these proteins in human biology and health and therefore the wealth of bibliographic information available for these proteins. Lectins in mammals, and more particularly in humans, have various functions that can be related to their localization (23). For instance, intracellular lectins are mostly involved in quality control of glycoprotein biosynthesis and intracellular trafficking (24). Calnexin and calreticulin maintain glycoproteins in the endoplasmic reticulum until they are correctly folded. Malectin directs misfolded proteins to the proteasome. P-type lectins transport new lysosomal enzymes to lysosomes (25). The quality control is also at play on the surface of cells where asialoglycoprotein receptors (ASGPR) on mammalian hepatocytes are involved in the turnover of serum glycoproteins. Through binding to endogenous glycans, cell surface lectins participate in cell–cell and cell–matrix interactions, such as L-selectin which directs lymphocyte homing from bloodstream to lymph nodes through an interaction with endothelial cells. Furthermore, human lectins are key players in innate immunity by recognizing non-self glycans on viruses, bacteria, parasites and fungi. Soluble lectins in serum activate a variety of defense mechanisms, from phagocytose to activation of the complement cascade (26). Lectins on immune cells possess intracellular signaling domains and are involved in activation and repression of immunity responses (27). This variety of functions and localizations is mirrored by the structural variety of CRDs, but also by a large range of architectures. Many lectins, such as some galectins, are composed of a single CRD, that can associate as dimers or oligomers, while others are part of complex multi-domain proteins that may be anchored to the plasma membrane to exert further signaling function(s) (28).

Finally, this update also spans the recent implementations improving the remote usability and interoperability of UniLectin. The application was containerised, an API (Application Programming Interface) was included and an RDF (Resource Description Framework) model was defined to allow for the development of a SPARQL interface. These functionalities not only facilitate cross-resource searches in glycoinformatics as advocated by the international GlySpace Alliance consortium cooperating towards open glycoscience (29), but are also a prerequisite for bridging with other bioinformatics initiatives applied to a broad range of -omics.

## Update of the Unilectin portal

### Update of UniLectin3D

The UniLectin3D module is manually updated with structures from the PDB on a monthly basis. The August 2023 version includes 2465 3D-structures from 629 different lectins, corresponding to an increase of approximately 12% compared to the 2021 version (16). The 3D structure visualisation interface was originally supported by LiteMol (30) using the 3D-SNFG plugin (31) to represent the glycan moiety. In 2021, LiteMol was upgraded to Mol* (32) in which the 3D-Symbol Nomenclature for Glycans (3D-SNFG) plugin is integrated. The selected view for the protein moieties is the ribbon representation.

### Update of LectomeXplore

**Figure 1.**
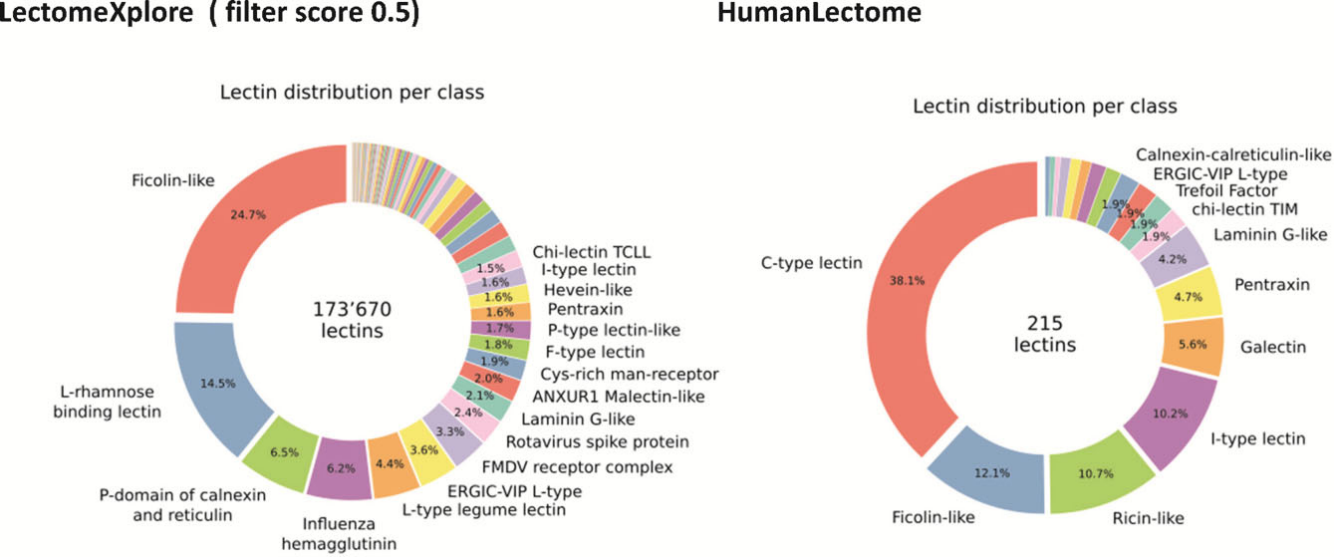
Comparison of the lectin distribution per class in LectomeXplore and HumanLectome. The class name and distribution percentage are only shown for slices with a lectin representation larger than 1.4%.

LectomeXplore is updated yearly by running the HMM profiles of 107 UniLectin classes on the UniProt and the NCBI-nr databases. The last release (July 2023) includes 1.4 M putative lectins, which represents a 20% increment compared to the 2021 version. 173 554 sequences are predicted with a high confidence score (>0.5) (Figure 1). Overall, the increment is well spread in all classes, with the exception of coronavirus spike proteins, which unsurprisingly increased by 40% in the last 2 years.

### Addition of new modules

The LectomeXplore approach brought the systematic detection of encoded lectins in whole translated genomes to the fore and several specific implementations sprung from there. This led to the creation of a few dedicated modules:

- MycoLec, collecting predicted fungal lectins in 1419 genomes of the MycoCosm database (33). Significant differences in the lectomes of translated genomes were identified and are in accordance with fungal taxonomic classes. Moreover, lectin occurrence could be correlated with ecological information available in the processed fungal species (21).
- TrefLec, collecting predicted β-trefoil lectins from complete proteomes. These proteins are popular for the design of new scaffolds with high symmetry in association with other domains. Further investigation of a predicted β-trefoil lectin occurring with an aerolysin domain led to confirm carbohydrate recognition and identify a pore forming toxin in a colony-forming micro-eukaryotic marine organism (20).

In addition, BiotechLec, a module describing the properties of commercially available lectins in relation with UniLectin information, was integrated as a guide for practical use (22).

## New technical features

### Site migration

UniLectin was originally hosted on a private cluster and recently transferred to our university environment (https://unilectin.unige.ch/ with https://unilectin.expasy.org/ as an alias) in order to gain control over the system. This was an opportunity to complement the production server with a development one so as to implement stricter testing procedures and apply systematic controls prior to updates. The website and its database were also containerised to enable rapid deployment on other systems, thereby increasing reusability and reproducibility.

The update process was performed in five steps: (i) configuration of a development server, (ii) deployment of the website in a Docker container on this new server, (iii) update of the MySQL database from version 5 to 8, (iv) update of the Apache version to 2.4 and the PHP version to 8.2, (v) fixing errors and warnings that emerged from version changes and the system update.

### Newly implemented API

The UniLectin platform now incorporates a RESTful API to offer both interactive and programmatic access to its open-source data (https://unilectin.unige.ch/api). The API provides two endpoints. The first one allows users to retrieve information related to UniLectin3D, while the second enables data extraction relative to predicted lectins from LectomeXplore. Several example queries, tailored to the anticipated needs of biologists, are provided in the interface page to guide users. Additionally, the interface is extensible by design, allowing for the easy addition of new queries and endpoints. Example queries guide users in selecting relevant programmatic query features, in particular the type(s) of data that can be fetched from each endpoint. When queries are submitted, results are returned in a standard JSON format. The API is implemented using Python and Flask, following a modular approach that enhances scalability and maintainability. Additionally, the API is containerized, running in a separate Docker container. This allows different tasks to be separated into individual containers. At present, the API offers two endpoints that correspond to distinct tables for UniLectin3D and for the predicted lectins of LectomeXplore where each column within these tables can be queried through the API. The architecture is designed for future expansion, allowing for additional endpoints and functionalities as the need arises.

### RDF ontology for UniLectin3D

An ontology-based mode of UniLectin3D was developed to provide a formal, explicit specification of a shared conceptualization of lectins and glycans. The ontology, called LectOn, is protein-centric by design and accounts for each protein potentially possessing one or multiple Carbohydrate Recognition Domains (CRDs), which in turn may bind to one or more glycan(s). Discussing the structure of the ontology is not in the scope of the present article. Yet, the scheme representing the first attempt to capture the complex relationships inherent in lectin-glycan interactions is shown in the dedicated section of UniLectin (https://unilectin.unige.ch/rdf/) where the LectOn wiki button prompts a detailed view of the LectOn features (unpublished work). This section also gives access to a SPARQL endpoint (SPARQL button) and a SPARQL query editor (SparqlSWEETS button).

A primary objective of developing this ontology is to ensure its compatibility and interoperability with existing ontologies in the field. To achieve this, we have established specific linkages between our ontology and others. For instance, glycans within our ontology are cross-referenced with the GlySTreeM ontology (34), destined to refine the representation of glycan structures. Similarly, in LectOn, proteins are mapped to their corresponding entries in the UniProt ontology. These linkages not only enrich the data but also make it easier to integrate LectOn into broader bioinformatics workflows.

## The human lectome database

A wide amount of information on animal lectins is available in literature (23,35,36). Information specific to human lectins can also be found (37), but not in a dedicated database. Even though LectomeXplore can be filtered with *Homo sapiens* in the species field, the resulting set of protein sequences is larger than expected from published reports (1741 with a score greater than 0.25 and 470 for a score >0.5). This is due in part to redundancy reflecting the content of the databases scanned by the LectomeXplore engine (e.g. only one human malectin is expected but seven entries are found in LectomeXplore). The ubiquity of lectin-like domains also creates ambiguity: several lectins adopt folds that are widespread but not necessarily associated with carbohydrate-binding properties. The most populated class of human lectin candidates in LectomeXplore is ‘ficolin-like’ (154 candidate lectins with a score >0.5) because the structure is shared with the frequently occurring fibrinogen domain. However, the biological and therapeutic interest of human lectins warrants the development of a database matching the reality of the human lectome. This entails a high level of curation to get around the listed pitfalls and, maybe more difficult, annotating ‘lectin-adequacy’, by a thorough analysis of literature.

Our initial choice for HumanLectome was to produce a protein-centric lectin functional description, which entails accounting for both the CRD and the entire protein levels. We therefore extracted information from Uniprot such as sequence, tissue localisation, AlphaFold Model and details of other domains with different functions (transmembrane, signalling, adhesion, enzymatic …) to cover the whole protein. Focus on the CRD is achieved by characterising the specificity or the glycan binding site and giving access to PDB structures or predicted models. As an exceptional case, the P22897 entry contains two functional CRDs, one C-type lectin and one Cys-Rich β-trefoil, specific for mannose and sulfated N-acetylgalactosamine, respectively, that are then described across two HumanLectome entries to remain consistent with the hierarchical classification of UniLectin.

### Data source and database construction

HumanLectome is a knowledgebase available at https://unilectin.unige.ch/humanLectome/. As a part of the UniLectin portal, it is based on technology previously mentioned for web-based database development (PHP and MySQL) as well as JavaScript libraries for interactive graphic tools. It is compatible with all devices and browsers.

The Human Lectome database was built by cross-checking sequences predicted in LectomeXplore with functional data extracted from different sources. In order to control the redundancy inherent to large sequence databases, an initial search was performed on the human genome of reference build 38 version 13. Only canonical sequences were kept (i.e. no isoforms), leading to the selection of 427 putative lectin domains associated with reference lectin coding genes. Extended curation was performed by cross-validating with (i) functional annotation in UniProt, (ii) Kurt Drickamer's ‘Genomic Resource for Animal Lectins’ at Imperial College (no longer online since 2020), (iii) recent review articles describing human lectins (37) as well as main classes of animal lectins, such as C-type lectins (38,39), galectins (28), I-type lectins (40,41). The post-curation final count reached a total of 215 entries (Figure 1).

From an implementation point of view, owing to the low number of entries and favouring the simplicity of maintenance, a single table for the 215 entries of the HumanLectome was built, with, in most cases, a single column per metric.

There are two levels of description for human lectins. Firstly, as tabulated summaries that include the protein name, several cross-reference ID(s) and classification details and secondly, as comprehensive information organised in different sections of a dedicated entry. Each of the summary entries links to the corresponding (if available) UniLectin3D and LectomeXplore pages, as well as to the HumanLectome individual detailed page. The individual HumanLectome page displays sequence, structure, ligand and expression data. It also provides genome-related data, and links to a variety of external resources (Supplementary Table S1 in supplemental information). Additionally, the NCBI mRNA sequence and CDS gene viewers were also integrated.

Note that Clusters of Differentiation (CDs) are detailed in HumanLectome based on previously defined categories of antigens found on the surface of leukocytes and other immune cells (42). Since many human lectins are involved in innate immunity, the mix of nomenclatures reflects the input of various scientific communities.

For 49% of the entries where one or more CRD X-ray structures are available from the Protein Data Bank (43), links to the corresponding UniLectin3D entries are provided and a visualisation is always suggested. In addition, all entries include several types of models. For example, the AlphaFold model, from AlphaFold2 (44) is accessible via Uniprot for the entire protein sequence, including the CRD and other domains. For more expert users, when no PDB is available, a link to SwissModel (45) allows the user to build the CRD by selecting the most appropriate models and controlling the homology modelling. In addition, ready-to-use CRD models are available using MODELLER version 10.0 (46), the most widely used comparative modelling software for 30 years. The advantage of the MODELLER software is its ability to use multiple models and its reasonable computational time. These 3D structural models were obtained from CRD sequences extracted from the whole lectin, by multiple alignment of its lectin class and comparison with known data from other species using Muscle software (47). For each CRD, several hundreds of structural models were generated and those with the minimum DOPE score (48) were retained. Depending on the structural supports available, 1–3 models were selected for each class. A cross-validation step between lectins with known structures was used to assess the feasibility of proposing lectin structural models according to their class. All sequences, alignments, supports and results can be downloaded independently. Quality assessment is provided by Ramachandran plots and MolProbity analysis (49).

### Content of the HumanLectome database and search options

The 215 HumanLectome entries are spread across 19 UniLectin classes (Figure 1 and Table 1). Roughly half of these have documented glycan binding properties and as such, were considered as reliably curated. A colour code was introduced to quickly reflect various levels of curation and confidence. Sufficient evidence for assigning a lectin role is shown with a green background and is strengthened by cross-links to matching entries in UniLectin3D and/or LectomeXplore (if only LectomeXplore then the green colour is paler). In contrast, when collected information was sparse, entries were considered as ‘lectin-like’, which means that hardly any carbohydrate-binding property is reported, despite possible cross-links to matching entries in UniLectin3D and/or LectomeXplore. The background colour is then light orange. Finally, very low evidence for carbohydrate-binding features as a red background for 78 entries.

**Table 1.** Content of HumanLectome - classes are ranked according to number of curated lectins

Class	Representative structure of lectin domain	Curated with 3D structures (number and names)	Curated without structures (number and names)	Low/very low evidence (number)
C-type lectin	1SL4	**24**	**13**	**45**
	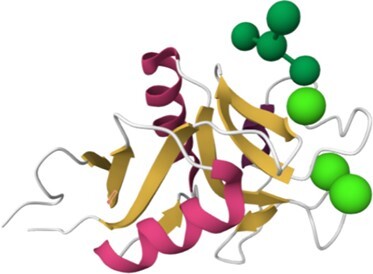	ASGPR-1, BDCA-2, BMPG, Collectin-K1, DC-SIGN, DC-SIGNR, DCIR, Dectin-2, E-selectin, Endo180, HIP-PAP, KLRB1, L-selectin, Langerin, MBP-C, MGL, Mincle, MMR, P-selectin, REG4, SP-D, SRCL, Tetranectin	Aggrecan, ASPGR-2, Brevican, Collectin-L1, Dectin-1, LSECtin, Prolectin, KLRD1, Kupffer Cell receptor, Layilin, Neurocan, REG3G, SP-A1, Versican	
I-type lectin	2DF3	**8**	**9**	**5**
	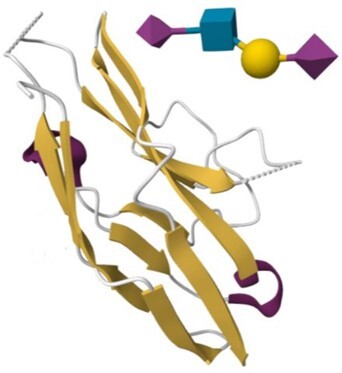	CD83, PLRA, SIGLEC-2, SIGLEC-3, SIGLEC-5, SIGLEC-7, SIGLEC-8, SIGLEC-15	NCAM-L1, SIGLEC-1, SIGLEC-4, SIGLEC-6, SIGLEC-9, SIGLEC-10, SIGLEC-11, SIGLEC-14, SIGLEC-16	
Galectin	1A3K	**7**		**5**
	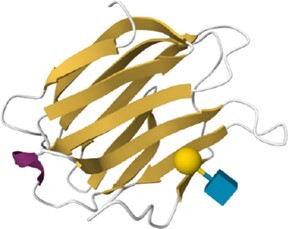	Galectin-1, Galectin-2, Galectin-3, Galectin-4, Galectin-8, Galectin-9, Galectin-13		
Ficolin-like	2JHK	**5**		**21**
	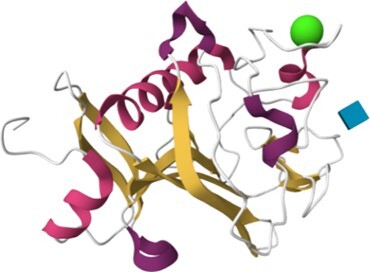	FIBCD1, H-ficolin, Intelectin 1, L-ficolin, M-ficolin		
Ricin-like	5AJO	**5**	**14**	**4**
	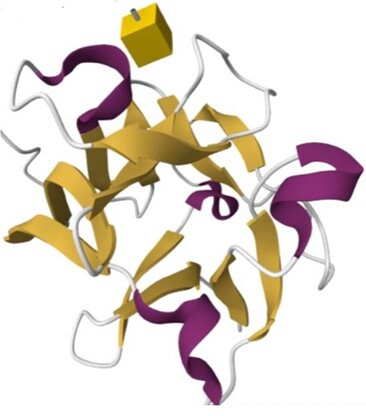	CBM13-ppGalNAc-T2, T-4, -T7, -T10, -T12	CBM13-ppGalNAc-T1, -T3, -T5, -T6, -TL6, -T8, -T9, -T11, -T13, -T14, -T15, -T16, -T17, -T18	
Chi-lectin TIM	4P8V	**3**		**1**
	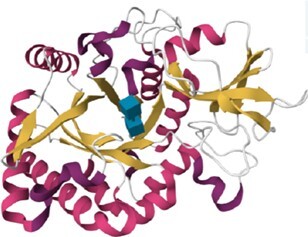	YKL-39, Hcgp-39, SI-CLP		
Trefoil factor	6V1C	**2**	**1**	**1**
	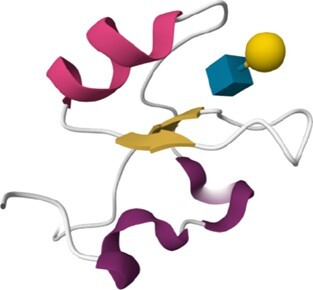	TFF1, TFF3	TFF2	
Pentraxin	1GYK	**2**		**8**
	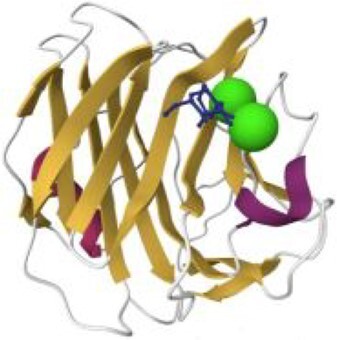	C-reactive protein, Serum amyloid P		
Jacalin-like	3VZE	**2**		
	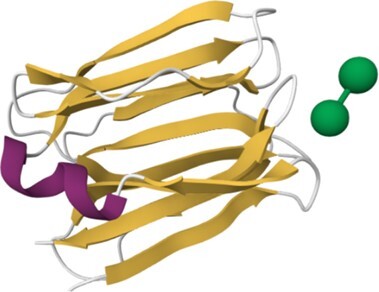	ZG16, ZG16B		
Calnexin-calreticulin-like	3POW	**1**	**2**	**1**
	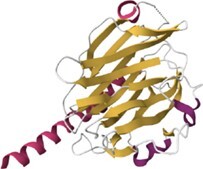	Calreticulin	Calnexin, Calmegin	
ERGIC-VIP L-type	4GKX	**1**	**2**	**1**
	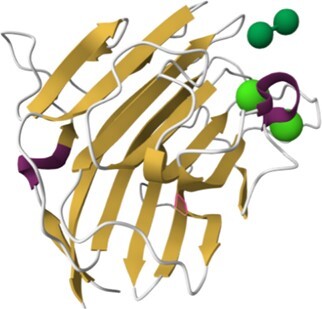	ERGIC-53	VIP36, VIPL	
P-type lectin	6Z30	**1**	**1**	
	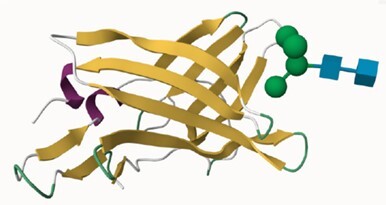	CI-MPR	CD-MPR	
P-type lectin-like	3AIH	**1**	**1**	
	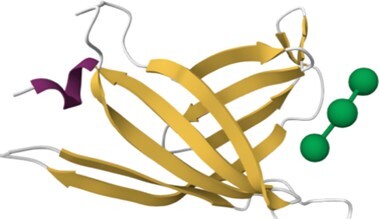	OS9	ERLEC1	
Laminin G-like	5IK5 (murine)	**1**		**8**
	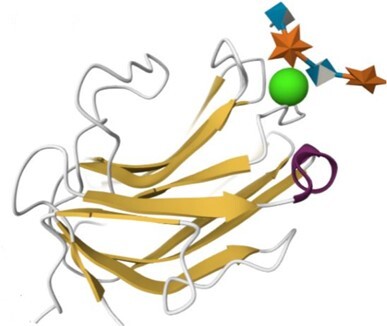	Laminin-α2		
Cys-rich man receptor	5XTW	**1**		
	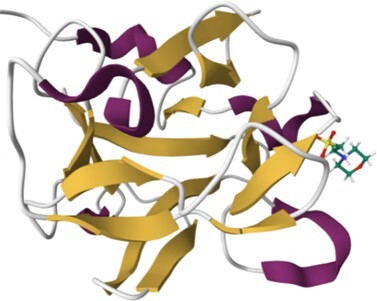	MMR		
F-box	2E33 (murine)		**1**	**2**
	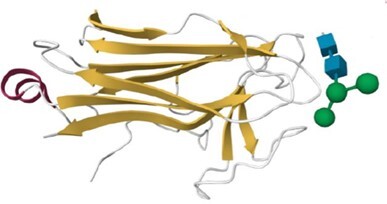		FBXO2-Fbs1	
Malectin	2K46 (Xenopus)		**1**	
	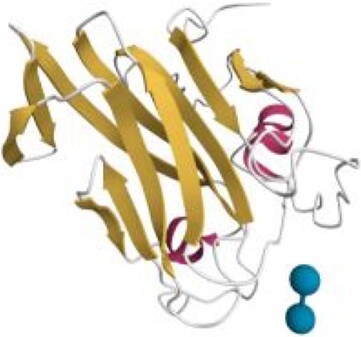		Malectin	
Tectonin				**1**
L-rha binding lectin				**3**

**Figure 2.**
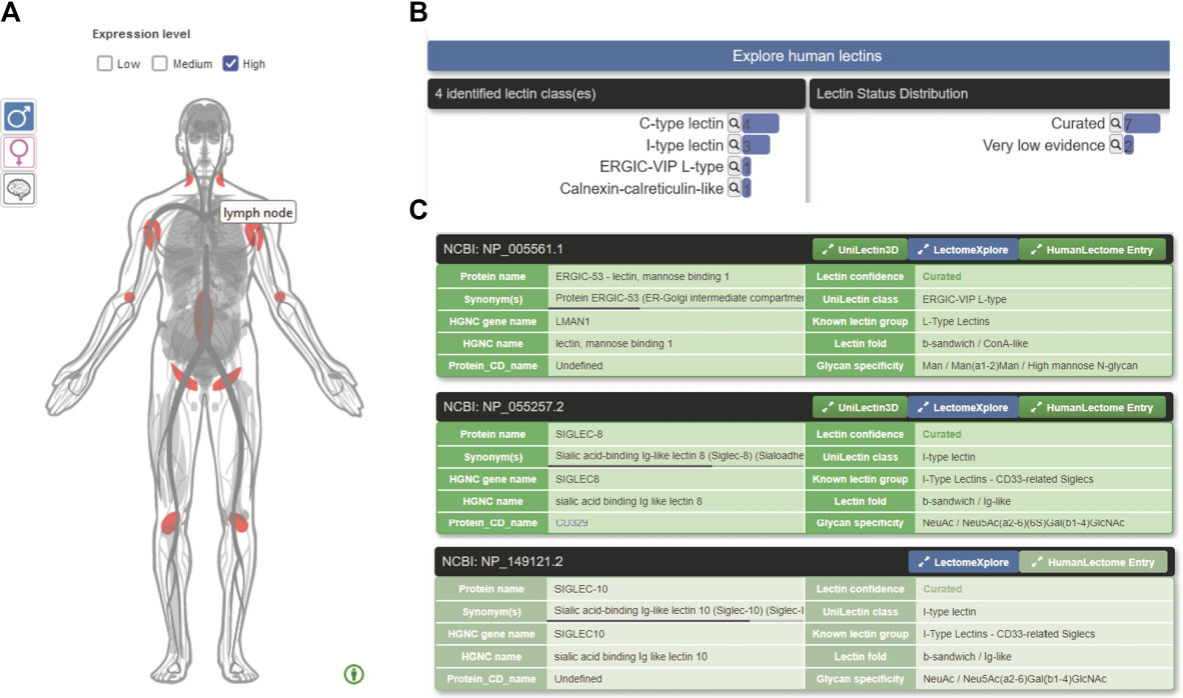
Example of search in Human Lectome. (**A**): selecting the lectins with high protein expression in the lymph nodes from the anatomogram, (**B**) class and lectin status distribution of the filtered lectins, (**C**) extract from some tabulated entries (3 entries among 9).

The database can be explored from the home page with three different options: (i) by tissues displayed in an anatomogram imported from the Gene Expression Atlas (50), (ii) by lectin type through the use of a sunburst and (iii) by lectin class on the same basis as in UniLectin3D. The anatomogram reflects lectin expression in pictured tissues, and search can be tuned according to recorded abundance (low/medium/high, high by default). For example, searching for lectins with high expression levels in lymph nodes results in nine lectins in four different classes (Figure 2). The curated entries contain two ubiquitous house-keeping intracellular lectins (ERCIG-53 and calreticulin) involved in quality control of glycoprotein synthesis, two C-type lectins (L-selectin and BDCA-2) and three I-type lectins (Siglec-8 and -10 and PILRA). The latter five lectins are known to be expressed at the surface of different cell types from the immune system (37).

A conventional search interface based on selection and combination of terms and filters is prompted by clicking on the ‘Explore human lectins’ button. The database can be queried by setting appropriate values in selected fields, such as, ‘curated’ for ‘Confidence status’ and ‘I-type’ for ‘Lectin class’. This example generates the list of 17 curated I-type lectin entries that are listed in Table 1.

Low and very low evidence lectins have been included despite the lack of carbohydrate recognition data or evidence of an active binding site. The C-type lectin domain contains a calcium-dependent CRD, however, many homologous proteins, associated in larger C-type lectin-like family, cannot coordinate with calcium and therefore do not have a canonical functional carbohydrate binding site (51,52). Among them, the secreted proteins of the regenerating gene (Reg) family that play a role in antimicrobial protection of the mammalian gut, human HIP-PAP (REGIII) (53) and REGIV (54), were demonstrated to perform their function through calcium independent polysaccharide binding. Our inclusive strategy has already been fruitful with ZG16B (Q96DA0) in the jacalin class. This protein was initially tagged as low evidence, but its status was moved to ‘curated’ following the recent demonstration that it binds bacterial carbohydrates of the oral microbiome (55).

### Curated data available from each entry page in HumanLectome

**Figure 3.**
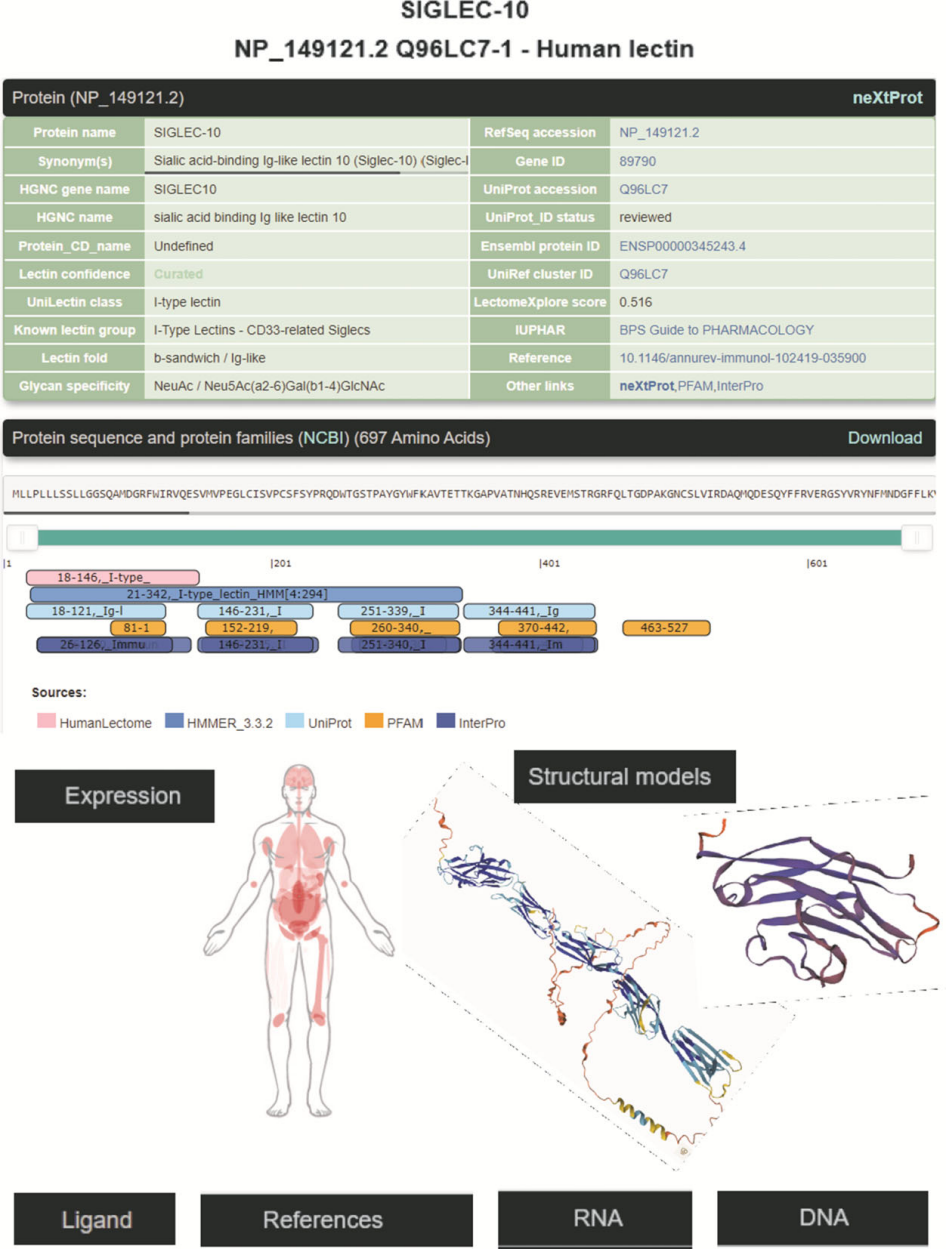
Selection of data available on the Siglec-10 entry page in HumanLectome.

The search strategies described above produce results in the form of summaries that provide access to the corresponding HumanLectome full entries. Figure 3 illustrates the case of Siglec10, a transmembrane protein highly expressed in peripheral blood leukocytes (56) for which no structural data is available (hence the pale green background). Siglec10 is referred to in main bioinformatics resources such as UniProt, RefSeq and most cross-references mentioned in Supplementary Table S1, as shown in the top of the entry. Curiously, unlike all other Siglecs (1–9) it is not associated with a CD number. Siglec10 contains only one CRD that is precisely defined in HumanLectome spanning position 18–146 (in pink) as a manual adjustment of the HMMER prediction from position 4 to 294 (in electric blue). In Protein Family databases such as InterPro, it is labeled as Immunoglobulin V-set domain and defined from position 26 to 126 (in dark blue). It also contains four immunoglobulin-like domains, well identified in InterPro, and three tyrosine-based motifs in its cytoplasmic tail.

No structure of Siglec10 was solved yet so that structural information in this entry is limited to predicted AlphaFold and homology-based models. In the absence of structures of Siglec-10 complexed with glycans, the specificity information is obtained from literature and displayed in the ‘glycan specificity’ window. Siglec-10 binds to both a-2,6- and a-2,3-sialoglycans as well as to GT1b ganglioside (57) and to GPI-anchored sialoprotein CD24 (58). Recent molecular modeling and NMR study rationalized this rather low specificity (59). In the Expression section, the high abundance of Siglec10 in bone marrow and lymphoid tissues according to the Human Protein Atlas is consistent with literature (58). Other sources remain fuzzier though each listing blood cells and lymph in higher values. At this stage, a lot of the references that follow are automatically extracted from cited sources but will increasingly account in the near future for cooperative input. Finally, via the NCBI viewers, the genomic context of Siglec10 is shown. RNA and DNA sequence data are made available for download.

Access to relevant and high 3D quality structural information is essential for many biotechnological and biopharmaceutical questions, and even for basic research. Having multiple versions of this information is also a definite added value. In this version of UniLectin, 49% of the CRDs are provided with crystal structures with one or more corresponding PDB references accessible from the entry page. Depending on the case, the PDB data describe a monomer, but also oligomers and in most cases with ligands represented in 3D SNFG symbols (60). To compensate for the lack of resolved structures, it was possible to generate a 3D model of all CRDs non-present in PDB by using the MODELLER software (46). The quality of the model was estimated using MolProbity with rmsd generally between 1.6 and 2.1 Ang. As an alternative source, the Swiss Model platform can be used for modelling through an interactive process that offers the possibility of selecting one or more supports from several crystal structures. Finally, the predicted AlphaFold2 structures are included for all structures since, contrary to the approaches above, it provides a model for the whole protein. In general, the CRD is well constructed, while the other domains have lower confidence scores. All of these structural models can be downloaded for further investigation or docking studies

## Conclusion and perspectives

Interest in elucidating glycan-protein interactions has significantly grown in recent years and the number of structural and functional data on lectins is growing fast with accompanying databases to collect and analyze them, such as Glycosmos lectins (61), ProCarbDB (62) that is no longer accessible, Dyonysus (https://www.dsimb.inserm.fr/DIONYSUS/), etc. However, our current lack of understanding of the glycocode is reflected in the ambiguity of the definition of binding specificity. Through its module collection, UniLectin offers a range of viewpoints in an attempt to put together sparse pieces of this puzzle. Experience gained from developing HumanLectome revealed the extent of inconsistencies of sources and confirmed the need for processing and curating data. More generally, UniLectin belongs to the Glyco@Expasy resource collection (63) along with GlyConnect (64) that describes glycoproteins with their glycans. Cross-links between UniLectin and GlyConnect are based on glycan substructure search and have been reestablished with a recent upgrade of glycan structure matching (65). Through these, a first approximation of the full complexes of a glycoconjugate with its corresponding glycan reader is made possible. Improved cross-talk between UniLectin and GlyConnect will also benefit from the current reappraisal of motifs and glycan epitopes within Glyco@Expasy.

Several challenges are awaiting UniLectin. Firstly, the integration of pathogen-related data; it should begin with reshaping SugarBindDB (66) into a UniLectin module. Secondly, the addition of finer structural information; the very rapid improvement in modeling/predicting protein folding is likely to soon allow for the inclusion of the oligomeric state of the CRD, or the positioning of metal ions in the structure, especially calcium ions that are crucial for the function of a large number of lectins. And last but not least, the fine prediction of glycan specificity for all curated entries that lack this information together with building models of the complex between CRD and predicted glycans, which is expected to be labour-intensive. Nonetheless, the recent release of several machine-learning based prediction methods (67–70) should adequately contribute to meeting this last goal.

The future of HumanLectome is to become increasingly encyclopedic and provide contextual information through our own curation but also through the contribution of experts from the scientific community of human lectins.

## Supplementary Material

gkad905_Supplemental_File

## Data Availability

The UniLectin portal is freely available at https://unilectin.unige.ch/.
