# Supplementary material for: HumanLectome, an update of UniLectin for the annotation and prediction of human lectins
Source: Nucleic Acids Res. 2023 Oct 27;52(D1):D1683–93. doi: 10.1093/nar/gkad905 (PMC10767822; doi:10.1093/nar/gkad905)
Supplement: gkad905_Supplemental_File [file gkad905_supplemental_file.docx]

**Supplemental Information**

**Table S1.** List of databases and websites that are cross-referenced or used in HumanLectome

| Database | URL | Information | Ref |
| --- | --- | --- | --- |
| UniProt | http://www.uniprot.org | Protein sequence information | (1) |
| NextProt | http://www.nextprot.org | Protein functional annotation | (2) |
| PDB | https://www.rcsb.org | 3D-structures of CRDs | (3) |
| PubMed | http://www.ncbi.nlm.nih.gov/pubmed | Bibliographic information |  |
| SwissModel | https://swissmodel.expasy.org | Modeling of CRDs | (4) |
| InterPro | https://www.ebi.ac.uk/interpro/ | Classification of domains | (5) |
| Expression Atlas | https://www.ebi.ac.uk/gxa/home | Anatomogram | (6) |
| Human Protein Atlas | https://www.proteinatlas.org | Information on expression | (7) |
| Genecards | https://www.genecards.org | Human Gene database | (8) |
| ProteomicsDB | https://www.proteomicsdb.org | Proteomics information | (9) |
| Glyconnect | https://glyconnect.expasy.org | Glycoprotein and glycans | (10) |
| HGNC_CD molecules | https://www.genenames.org/data/genegroup/#!/group/471 | CD in human gene nomenclature |  |
| Ensembl | The human genome assembly | Human genome assembly | (11) |
| NCBI | https://www.ncbi.nlm.nih.gov | Gene viewer |  |
| Dyonysus | https://www.dsimb.inserm.fr/DIONYSUS/ | Carbohydrate binding interface |  |
| Glycosmos | https://glycosmos.org/ | Integrated glycoscience | (12) |
| Glyco@Expasy | https://glycoproteome.expasy.org/glycomics-expasy/ | glycoinformatics resources | (13) |

1. UniProt, C. (2019) UniProt: a worldwide hub of protein knowledge. *Nucleic Acids Res*, **47**, D506-D515.

2. Zahn-Zabal, M., Michel, P.A., Gateau, A., Nikitin, F., Schaeffer, M., Audot, E., Gaudet, P., Duek, P.D., Teixeira, D., Rech de Laval, V. *et al.* (2020) The neXtProt knowledgebase in 2020: data, tools and usability improvements. *Nucleic Acids Res*, **48**, D328-D334.

3. Berman, H.M., Westbrook, J., Feng, Z., Gilliland, G., Bhat, T.N., Weissig, H., Shindyalov, I.N. and Bourne, P.E. (2000) The Protein Data Bank. *Nucleic Acids Res*, **28**, 235-242.

4. Waterhouse, A., Bertoni, M., Bienert, S., Studer, G., Tauriello, G., Gumienny, R., Heer, F.T., de Beer, T.A.P., Rempfer, C., Bordoli, L. *et al.* (2018) SWISS-MODEL: homology modelling of protein structures and complexes. *Nucleic Acids Res*, **46**, W296-W303.

5. Paysan-Lafosse, T., Blum, M., Chuguransky, S., Grego, T., Pinto, B.L., Salazar, G.A., Bileschi, M.L., Bork, P., Bridge, A., Colwell, L. *et al.* (2023) InterPro in 2022. *Nucleic Acids Res*, **51**, D418-D427.

6. Moreno, P., Fexova, S., George, N., Manning, J.R., Miao, Z., Mohammed, S., Munoz-Pomer, A., Fullgrabe, A., Bi, Y., Bush, N. *et al.* (2022) Expression Atlas update: gene and protein expression in multiple species. *Nucleic Acids Res*, **50**, D129-D140.

7. Sjostedt, E., Zhong, W., Fagerberg, L., Karlsson, M., Mitsios, N., Adori, C., Oksvold, P., Edfors, F., Limiszewska, A., Hikmet, F. *et al.* (2020) An atlas of the protein-coding genes in the human, pig, and mouse brain. *Science*, **367**.

8. Stelzer, G., Rosen, N., Plaschkes, I., Zimmerman, S., Twik, M., Fishilevich, S., Stein, T.I., Nudel, R., Lieder, I., Mazor, Y. *et al.* (2016) The GeneCards Suite: From Gene Data Mining to Disease Genome Sequence Analyses. *Curr Protoc Bioinformatics*, **54**, 1 30 31-31 30 33.

9. Lautenbacher, L., Samaras, P., Muller, J., Grafberger, A., Shraideh, M., Rank, J., Fuchs, S.T., Schmidt, T.K., The, M., Dallago, C. *et al.* (2022) ProteomicsDB: toward a FAIR open-source resource for life-science research. *Nucleic Acids Res*, **50**, D1541-D1552.

10. Alocci, D., Mariethoz, J., Gastaldello, A., Gasteiger, E., Karlsson, N.G., Kolarich, D., Packer, N.H. and Lisacek, F. (2019) GlyConnect: Glycoproteomics goes visual, interactive, and analytical. *J Proteome Res*, **18**, 664-677.

11. Martin, F.J., Amode, M.R., Aneja, A., Austine-Orimoloye, O., Azov, A.G., Barnes, I., Becker, A., Bennett, R., Berry, A., Bhai, J. *et al.* (2023) Ensembl 2023. *Nucleic Acids Res*, **51**, D933-D941.

12. Yamada, I., Shiota, M., Shinmachi, D., Ono, T., Tsuchiya, S., Hosoda, M., Fujita, A., Aoki, N.P., Watanabe, Y., Fujita, N. *et al.* (2020) The GlyCosmos Portal: a unified and comprehensive web resource for the glycosciences. *Nat Methods*, **17**, 649-650.

13. Mariethoz, J., Alocci, D., Gastaldello, A., Horlacher, O., Gasteiger, E., Rojas-Macias, M., Karlsson, N.G., Packer, N. and Lisacek, F. (2018) Glycomics@ExPASy: Bridging the gap. *Mol Cell Proteomics*.
